# Structure and functional impact of seed region variant in MIR-499 gene family in bronchial asthma

**DOI:** 10.1186/s12931-017-0648-0

**Published:** 2017-09-08

**Authors:** Eman A. Toraih, Mohammad H. Hussein, Essam Al Ageeli, Eman Riad, Nouran B. AbdAllah, Ghada M. Helal, Manal S. Fawzy

**Affiliations:** 1 Genetics Unit, Department of Histology and Cell Biology, Faculty of Medicine, Suez Canal University, Ismailia, P.O. 41522 Egypt; 2 Pulmonologist, Ministry of Health, Cairo, Egypt; 3 Department of Clinical Biochemistry (Medical Genetics), Faculty of Medicine, Jazan University, Jazan, Saudi Arabia; 4 Department of Chest Diseases and Tuberculosis, Faculty of Medicine, Suez Canal University, Ismailia, P.O. 41522 Egypt; 5 Department of Pediatrics, Faculty of Medicine, Suez Canal University, Ismailia, Egypt; 6 Department of Medical Biochemistry, Faculty of Medicine, Mansoura University, Mansoura, Egypt; 7 Department of Medical Biochemistry, Faculty of Medicine, Suez Canal University, Ismailia, P.O. 41522 Egypt; 8 Department of Medical Biochemistry, Faculty of Medicine, Northern Border University, Arar, Kingdom of Saudi Arabia

**Keywords:** Asthma, Egyptians, miR-499a, miR-499b, Polymorphism, Airway hyper-responsiveness, qRT-PCR

## Abstract

**Background:**

Small non-coding RNAs (microRNAs) have been evolved to master numerous cellular processes. Genetic variants within microRNA seed region might influence microRNA biogenesis and function. The study aimed at determining the role of microRNA-499 (MIR-499) gene family polymorphism as a marker for susceptibility and progression of bronchial asthma and to analyze the structural and functional impact of rs3746444 within the seed region.

**Methods:**

Genotyping for 192 participants (96 patients and 96 controls) in the discovery phase and 319 subjects (115 patients and 204 controls) in the replication phase was performed via Real Time-Polymerase Chain Reaction technology. Patients underwent the methacholine challenge test and biochemical analysis. Gene structural and functional analysis, target prediction, annotation clustering, and pathway enrichment analysis were executed. Predicted functional effect of rs37464443 SNP was analyzed.

**Results:**

miR-499 gene family is highly implicated in inflammation-related signaling pathways. Rs374644 (A > G) in MIR499A and MIR499B within the seed region could disrupt target genes and create new genes. The G variant was associated with high risk of developing asthma under all genetic association models (G versus A: OR = 3.27, 95% CI = 2.53–4.22; GG versus AA: OR = 9.52, 95% CI = 5.61–16.5; AG versus AA: OR = 2.13, 95% CI = 1.24–3.46; GG + AG versus AA: OR = 4.43, 95% CI = 2.88–6.82). GG genotype was associated with poor pre-bronchodilator FEV_1_ (*p* = 0.047) and the worst bronchodilator response after Salbutamol inhalation, represented in low peaked expiratory flow rate (*p* = 0.035).

**Conclusions:**

miR-499 rs3746444 (A > G) polymorphism was associated with asthma susceptibility and bronchodilator response in Egyptian children and adolescents. Further functional analysis is warranted to develop more specific theranostic agents for selecting targeted therapy.

**Electronic supplementary material:**

The online version of this article (10.1186/s12931-017-0648-0) contains supplementary material, which is available to authorized users.

## Background

Bronchial asthma is a chronic heterogeneous respiratory disease that is characterized by airway inflammation, recurring bronchial obstruction, and airway hyper-responsiveness [1, 2]. Most common histopathological features are inflammatory cell infiltration, sub-basement fibrosis, smooth muscle hypertrophy, mucus hypersecretion, injury to epithelial cells, and angiogenesis [3, 4]. Treatment with anti-inflammatory drugs or bronchodilators usually improves some of these features. Nevertheless, therapeutic response relies on the interplay between environmental exposure and genetic background [2]. Despite several advances over the past decades in understanding the underlying mechanisms involved in the disease, there are no current satisfactory strategies for the cure or prevention of long-term decline in pulmonary function [5]. Given the significant morbidity and burden of childhood asthma worldwide, better therapeutic modalities are mandatory to counteract the development and progression of the disease. In prior studies, a sizeable proportion of genetic influence existed, ranging from 35 to 95% for asthma and 30 to 66% for bronchial hyper-responsiveness [6]. Genome-wide association studies provide evidence for multiple novel loci associated with the disease. However, the exact maestro fine tuning these putative genes is still uncovered.

In early 90’s, the presence of small non-coding RNAs (ncRNAs) was discovered in the mammalian genome [7]. These microRNAs are transcriped via specific cellular machinery to form short single-stranded mature RNAs of 19–24 nucleotides long. They function by complementary base pairing with mRNA targets, leading to its degradation or translational repression [8]. They are estimated to modulate gene expression of 60% of protein-coding genes, and to regulate many cellular processes; including proliferation, apoptosis, immunomodulation, stress response, and angiogenesis [9]. As a result the focus of human genome studies has witnessed a shift from mRNAs to ncRNAs as major key players in human disorders. Currently, there are emerging opportunities for targeting these disruptions of ncRNAs using novel therapeutic approaches. Some strategies aimed to increase the levels of abnormally down-regulated microRNAs via epigenetic drugs as DNA demethylating agents and histone deacetylase (HDAC) inhibitors or by replacement of miRNAs using virus delivery systems [10]. On the other hand, over-expressed microRNAs in human diseases can be sequestered by anti-miRNA oligonucleotides, miRNA sponges, miRNA masking and small molecule inhibitors [8].

**Fig. 1:**
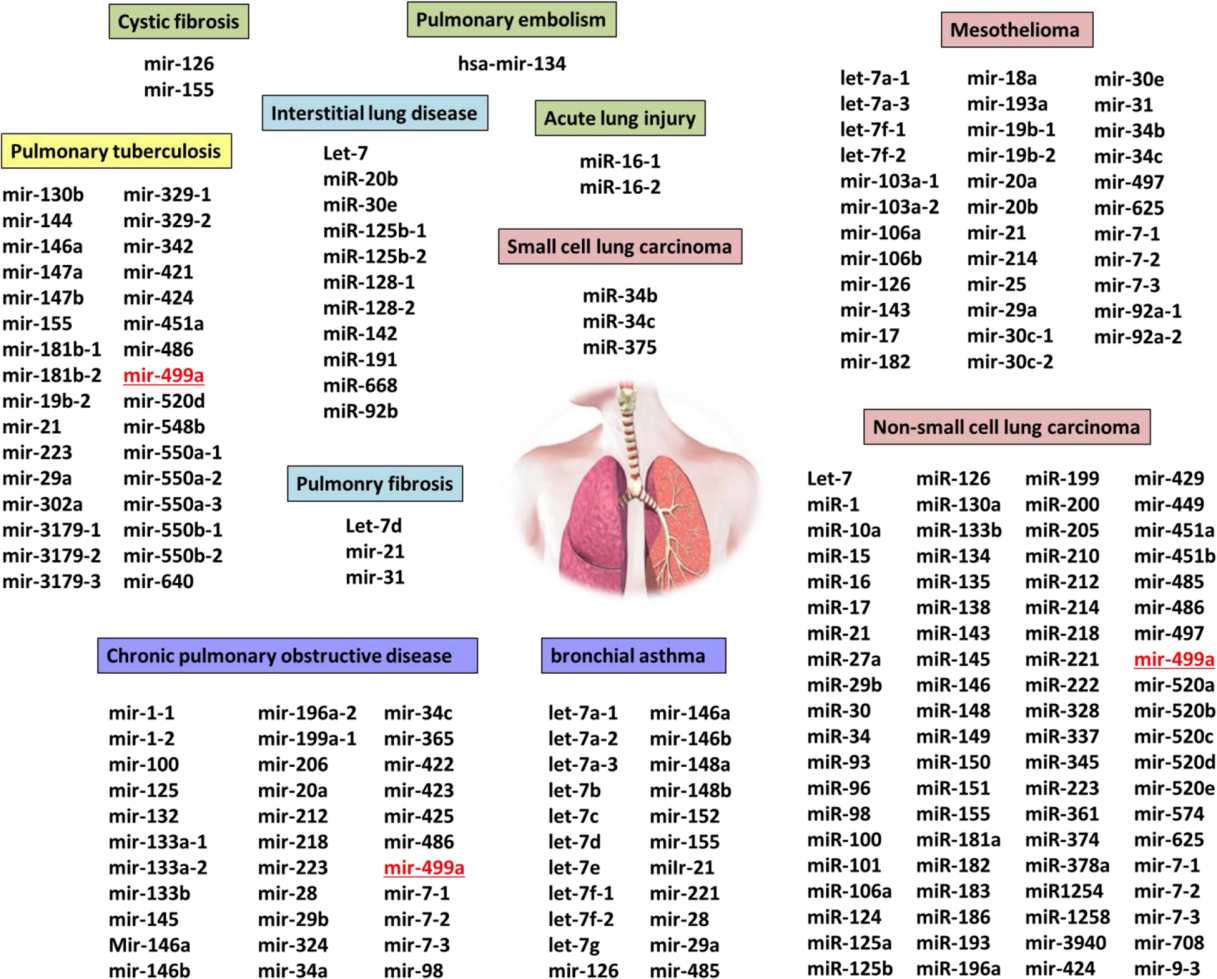
MicroRNA involved in pathological lung conditions. MicroRNAs were found to be associated with several respiratory disorders by prior high-throughput and experimentally validated studies. One of them, miR-499a was significantly associated with pulmonary tuberculosis, chronic pulmonary obstructive disease, and non-small cell lung cancer. Data source: Literature surveying and miRTarBase for experimentally validated miRNA-target interactions database (http://mirtarbase.mbc.nctu.edu.tw/)

In silico analysis and surveying the literature revealed the deregulation of microRNAs in various pulmonary diseases (Fig. 1). Several lines of evidence suggest a key role for hsa-microRNA-499a (miR-499a) in modulating the immune response, cell proliferation, apoptosis, neuromuscular regulation and neoangiogenesis [11, 12]. Exploring gene targets of miR-499a by computational tools identified inflammatory-related gene targets, including IL-13 and Il-23, which represent important mediators in asthma KEGG pathway [ID hsa05310] (microRNA.org). A single nucleotide polymorphism (SNP), rs3746444 (A > G), is located in the seed sequence of miR-499a-3p, a region essential for miRNA-mediated silencing mechanism. SNPs within mRNA-binding site of miRNAs may influence mRNA gene set, target binding ability, or pre-miRNA maturation process, which in turn, could alter the susceptibility to develop human diseases. The rs3746444 MIR-499a SNP was found to be associated with higher risk of rheumatoid arthritis [12, 13], coronary artery disease [14], Behcet’s disease [15], and ankylosing Spondylitis [16]. Hence, the current study was conducted to investigate the association between rs3746444 polymorphism and susceptibility to asthma disease in children and adolescents, and to further assess computationally its impact on the clinical outcome and bronchial hyper-responsiveness (BHR) in a group of Egyptian asthmatic children and adolescents.

## Methods

### Structural gene analysis

Genetic analysis of MIR499A gene was performed using Genecards.org, Ensembl.org, and NCBI. DNA and RNA sequences were retrieved from miRBase.org. Multiple sequence alignment and phylogenetic tree construction were implemented to identify similarity regions in different species by Ensembl.org and with its cluster gene MIR499B by Rcoffee v11.0, a specific method for non-coding RNA [17]. Analysis by PolymiRTS Database v3.0 (Polymorphism in microRNAs and their TargetSites) was conducted to predict SNPs and INDELs in the whole gene (http://compbio.uthsc.edu/miRSNP/home.php) [18]. Variant calls in the chromosomal region were obtained from Ensembl.org and UCSC via usegalaxy.org platform.

### In silico target gene prediction

Next, list of computationally predicted and experimentally validated gene targets (Additional file 1: Table S2) were retrieved from multiple databases including DIANA-microT-CDS v5.0 (http://diana.imis.athena-innovation.gr/DianaTools/index.php?r=microT_CDS/index), miRBase (http://www.mirbase.org/), TargetScanHuman v6.2 (http://www.targetscan.org/), miRDB (http://mirdb.org /miRDB/), miRNAMap v2.0 (http://mirnamap.mbc.nctu.edu.tw/), and DIANA-TarBase v7.0 algorithm (http://diana.imis.athena-innovation.gr/DianaTools/index.php?r=tarbase/index) databases. Result intersection and statistical validation were performed as described previously [12].

### Functional annotation clustering and pathway enrichment analysis

Functional analysis of the gene list was done via Diana-miRPath v3.0 software for gene ontology (GO) terms and Kyoto encyclopedia of genes and genomes (KEGG) pathways [19]. Fisher’s exact test/hypergeometric statistical test was applied at microT-CDS threshold of 0.8 and *P* value threshold at 0.05. Gene Ontology (GO) system of classification was carried out to interpret the target gene sets of miR-499a-3p, miR-499a-5p, miR-499b-3p, and miR-499b-5p based on their functional characteristics. The attributes of targets were classified and ranked in the context of biological process, molecular function, or cellular component. Further comparison of functional categories between miR-499a and miR-499b targets was conducted by miRpair2Go web-platform using two combined miRNA target prediction methods (TargetScan and miRanda) and moderate hierarchial filtering level (http://compbio.uthsc.edu/miR2GO/help.php) [20].

### Predicted functional effect of rs37464443 SNP

Distribution of SNPs in miR-499 was analyzed via miRdSNP database (http://mirdsnp.ccr.buffalo.edu/): a database of disease-associated SNPs to identify the spatial relationship of the miRNA with target sites on the 3’UTR of human genes and to further explore the molecular mechanism of gene deregulation at the post-transcriptional level [21]. The predicted functional impact of miR-499 rs3746444 variant was performed using miRmut2Go (http://compbio.uthsc.edu/miR2GO) to analyze the changes of target genes caused by miRNA mutations and view the functional impacts of these changes in the context of comparative functional GO analysis using the same filters described in our prior work [9]. Advanced gene set enrichment analysis was employed using the Gene Trail program with multiple testing correction via false discovery rate (FDR) estimation and significant level at 0.05 (https://genetrail2.bioinf.uni-sb.de/) [22]. In addition, mfold RNAfold (http://unafold.rna.albany.edu/?q=mfold) and KineFold web-servers (http://kinefold.curie.fr/) were conducted to predict the secondary structures of RNA sequence in A and G alleles.

### Study participants

A total of 211 asthmatic patients and 300 controls (age range 3 to 18 years old) were enrolled in the study. In the discovery stage: the study participants were composed of 96 patients and 96 controls. Patients were obtained from the Pediatrics outpatient clinic of Suez Canal University Hospital (SCUH), Ismailia, and Chest and tuberculosis Department, Kasr Al-Ainy Hospital, Giza. Whereas, in the replication phase: other independent cohorts of 115 patients and 204 controls were recruited from SCUH, Ismailia. They were diagnosed and assessed according to the Global Initiative for Asthma (GINA) guidelines [23]. A thorough clinical assessment was performed for determining disease severity, therapeutic history, and co-morbidities as previously described [2]. Controls had no history of wheezes, atopy, or any other respiratory diseases. Chest X-ray was done for participants to exclude concurrent chest disease. Body mass index (BMI) percentile of patients and controls was estimated and adjusted for age and sex using an online pediatric calculator (http://www.quesgen.com/BMIPedsCalc.php) [24]. Sexual maturity rating was determined based on Tanner classification [25]. The study was conducted in accordance with the guidelines in the Declaration of Helsinki and had the approval of the Ethics Committee of Faculty of Medicine, Suez Canal University. Informed consent was obtained from participants’ parents.

### Spirometry and methacholine challenge test

Baseline pulmonary function test assessment was done using an electronic Spirometer (BTL-08 Spiro Pro system; BTL) with a valve-spacer device following the guidelines of the American Thoracic Society/European Respiratory Society (ATS/ERS) [26]. Baseline lung parameters were documented [27]. Repeated post-bronchodilator forced Spirometry was performed 15 min after administering a 400 μg dose of inhaled Salbutamol (Ventolin; GlaxoSmithKline). BDRBASE, change in FEV_1_ as a percent of baseline forced expiratory volume at 1 s (FEV_1_), was calculated with the following equation [= ((postbronchodilator FEV_1_ − prebronchodilator FEV_1_) / prebronchodilator FEV_1_) × 100] [27]. Additionally, bronchoconstriction provocation via methacholine challenge test (MCT) was done to assess bronchial hyper-responsiveness. Methacholine solution, mixed with saline with the following gradient doses (0.06, 0.125, 0.25, 1, 2, 4, 8, 16 mg/ml), were aerosolized using a nebulizer attached to an air compressor at 5 min interval. Progressive increase in concentration was used, until the patient encountered a significant worsening in lung function, with a drop in FEV1 of 20% or more. BHR was categorized according to the American Thoracic Society guidelines with positive cutoff value defined as a PC20 below 8 mg/ml [28, 29].

### Biochemical analysis

Blood samples were collected in EDTA Vacutainers. Absolute eosinophil count (AEC) was calculated using the Coulter Counter. Total IgE was measured by enzyme-linked immunosorbant assay (ELISA). Absolute esinophilic count <0.1 × 10^3^/μl and total IgE concentrations <90 IU/ml were defined as normal [30].

### Genotyping of seed region variant

Genomic DNA was purified from whole blood using QIAamp DNA Blood Mini kit (Catalog No. 51104; Qiagen) following the manufacturer’s protocol. Extracted DNA purity and concentration were assessed by NanoDrop ND-1000 (NanoDrop Technologies, Inc. Wilmington, DE, USA). Genotyping for the hsa-miR-499a (rs3746444) was assayed using Real-Time polymerase chain reaction (RT-PCR) allelic discrimination technology. PCR reactions were run blindly in duplicates in a 25-μl final volume containing 20 ng genomic DNA, TaqMan Universal PCR Master Mix, No UNG (4440043), and TaqMan SNP Genotyping Assay Mix (assay ID C_2142612_30, Applied Biosystems) with 100% concordance rate for genotype calls. Appropriate controls were used in each reaction. PCR amplification was done using StepOne™ Real-Time PCR System (Applied Biosystems, USA) [12]. Allelic discrimination was called by the SDS software version 1.3.1 (Applied Biosystems).

### Statistical analysis

Statistical analysis was performed using PCORD v.5.0, R programming language and the *“*Statistical Package for the Social Sciences (SPSS) for windows*”* software, version 22. Allele and genotype frequencies and carriage rate were calculated as previously described [31]. The Hardy-Weinberg equilibrium was estimated using the Online Encyclopedia for Genetic Epidemiology (OEGE) software (http://www.oege.org/software/hwe-mr-calc.shtml) and tested for goodness of fit by chi square test. Genotype and allele frequencies were compared between asthmatic patients and control subjects using the chi-square test. Adjusted odds ratios (OR) with a 95% confidence interval (CI) by logistic regression analysis were calculated for multiple genetic association models. Data distribution was checked by the Kolmogorov-Smirnov test. Appropriate data presentation and test were used for comparison between groups. A two-tailed *P-*value of 0.05 was considered statistically significant. Both ordination and two-way agglomerative hierarchal clustering techniques were applied to the data for multivariate analysis [32].

## Results

### Structural gene analysis and comparative genomics

**Fig. 2:**
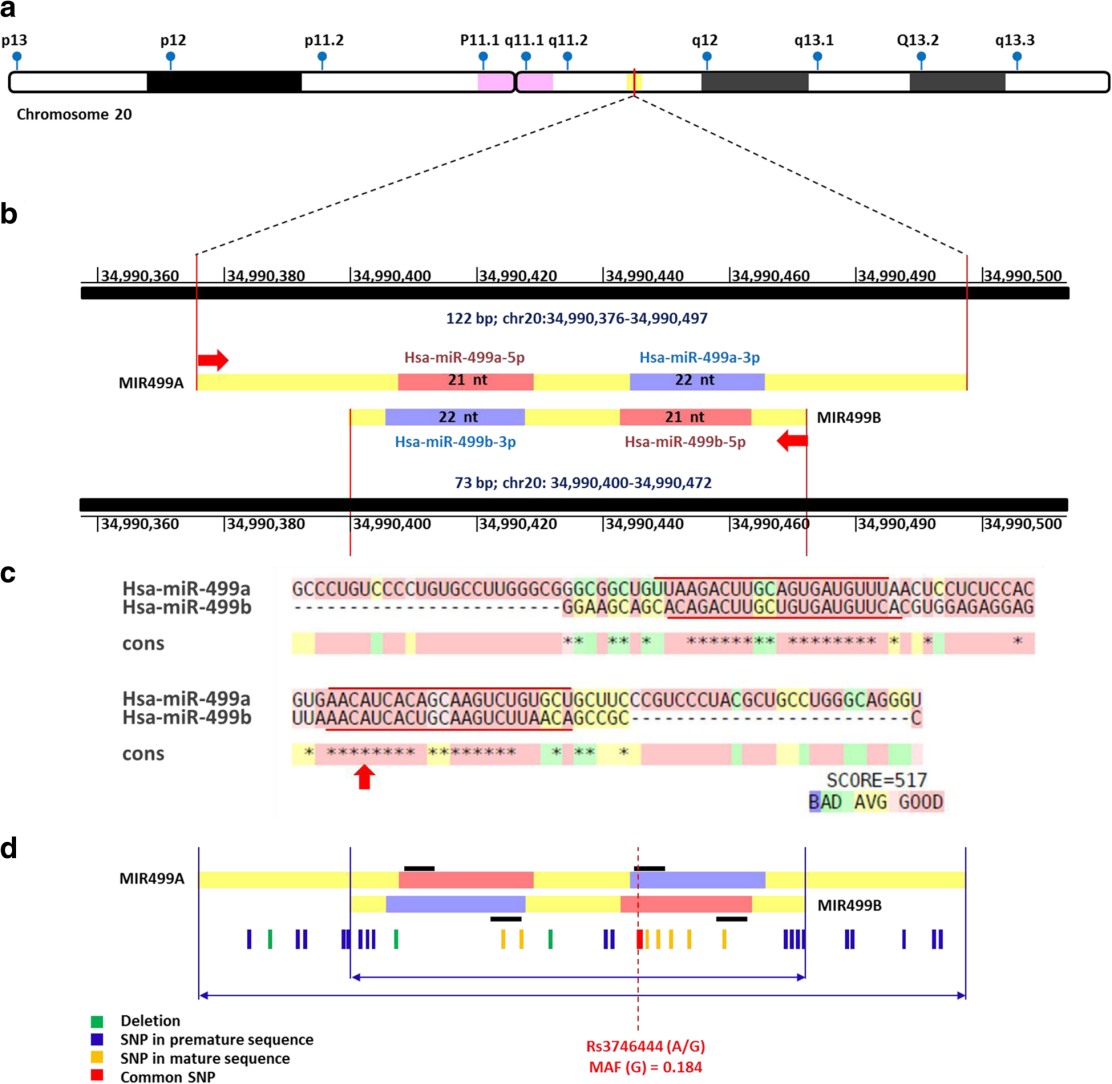
Genomic structure of human MIR-499A gene. (**a**) Chromosomal location along the long arm of chromosome 20q11.22. (**b**) MIR499A gene (ENSG 00000207635) is located on the forward strand spanning 122 bp long (Genomic coordinates at 20:34,990,376–34,990,497; according to the Human Genome Assembly GRCh38, release annotation 108). Whereas, MIR499B gene (ENSG00000283441) is located on the other strand spanning 73 bp in length (20: 34,990,400–34,990,472). The direction of miRNA transcription is shown by red arrows. MiR-499a is transcribed from the forward strand. MiR-499b is transcribed from the reverse strand. Each miRNA has two embedded mature forms; one at the 5′ end (5p) in pink (21 nucleotides), the other at 3′ end in blue (22 nucleotides). (**c**) High conservation shown by the pairwise alignment produced using Rcoffee platform. Positions of mature forms are shown by the red line. The red arrow shows the studied SNP in the study (rs3746444; A/G). (**d**) Variant analysis of the MIR499A gene. A total of 30 variants (3 deletion and 27 SNPs), were identified. Of them, 19 variants are overlapped with MIR499B gene variants. Seed region is represented by a black bar at the 5′ end of each mature miRNA

**Fig. 3:**
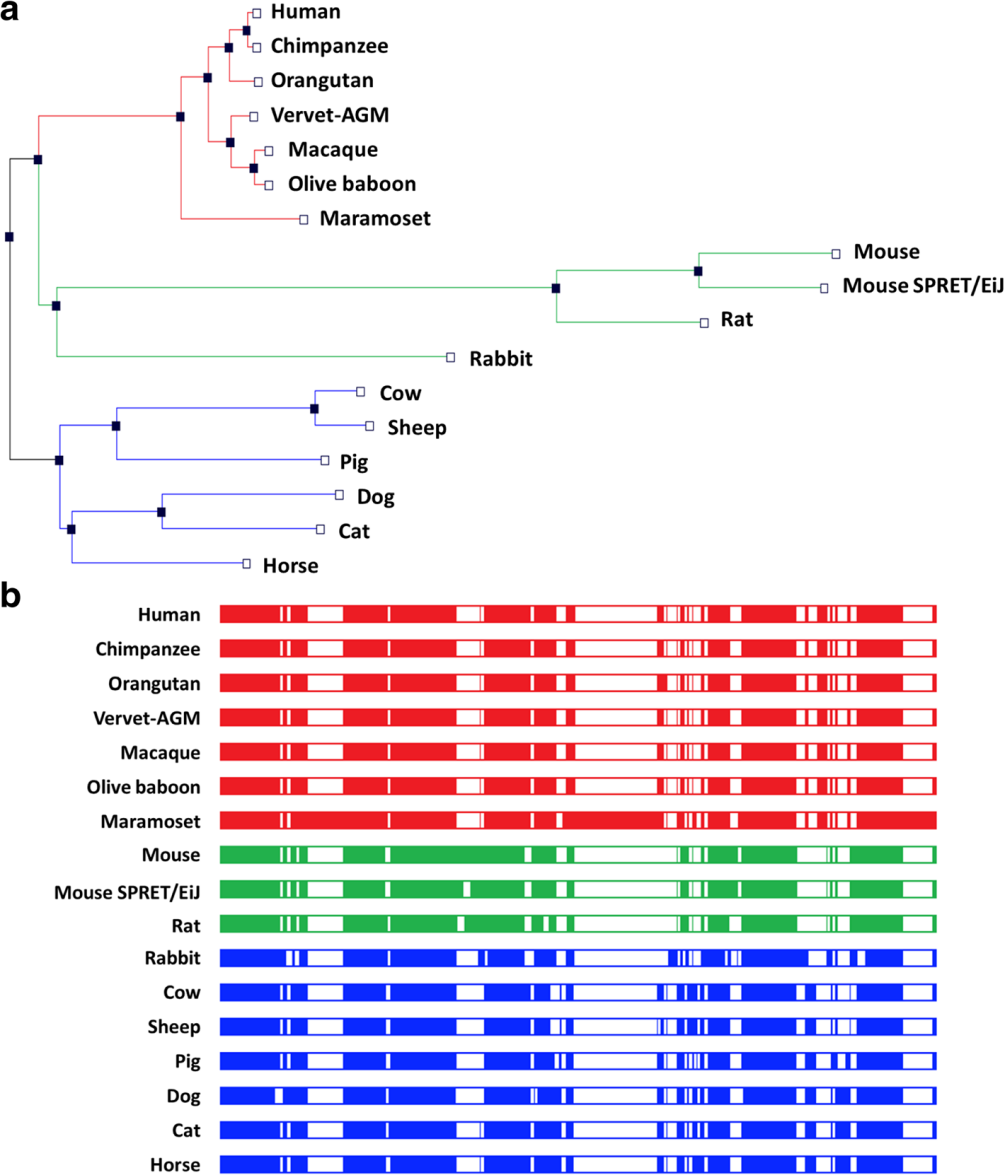
Human MIR499A shows high conservation across different species. (**a**) Phylogenetic tree showing the degree of similarity among different species. Close similarities exist with higher primates than remote species. (**b**) Multiple sequence alignment was done via Ensemble.org. The figure shows that miR-499a is existed in a highly conserved genomic locus

Human miR-499a gene (MIR499A*;* ENSG 00000207635) is located along the long arm of chromosome 20q11.22 spanning 122 bp long (Genomic coordinates at 20:34,990,376–34,990,497 on the forward strand; according to the Human Genome Assembly GRCh38, release annotation 108). MIR499A gene exists within intron 19 of myosin, heavy chain 7B, cardiac muscle, beta *MYH7B* gene and overlapping MIR499B gene (ENSG00000283441; 73 bp in length; 20: 34,990,400–34,990,472 on the reverse strand) (Fig. 2). Similar orthologs are present in other species; mouse MIR-499 was also mapped to intron 19 of the *Myh7b* gene on chromosome 2 (*Mus musculus*; 2:155,622,880–155,622,958 (+); GRCm38). Whereas rat MIR-499, existed on chromosome 3 (*Rattus norvegicus*; 3:151,138,862–151,138,926 (+); Rnor6.0). Multiple sequence alignment showed MIR499A gene to display a high level of conservation throughout 17 mammalian species (Fig. 3).

MIR499A consists of a single exon that encodes for a single transcript (MI0003183) of 122 bp long. Whereas, its clustered MIR499B forms a shorter transcript of 73 bp long from the reverse strand. Variant analysis of MIR499A identified the presence of 30 non-coding transcript exon variants (27 SNPs and 3 deletions) in MIR499A including 19 overlapping variants (17 SNPs and 2 deletions) existed in MIR499B. All variants were rare except rs3746444 (A/G alleles) at the position 20:34,990,448 (*GRCh38*) with minor allele frequency (MAF) of 0.184. The rs3746444 variant exists in the seed region of miR-499a-3p; AC[A/G]UCAC.

### In silico target gene prediction

**Fig. 4:**
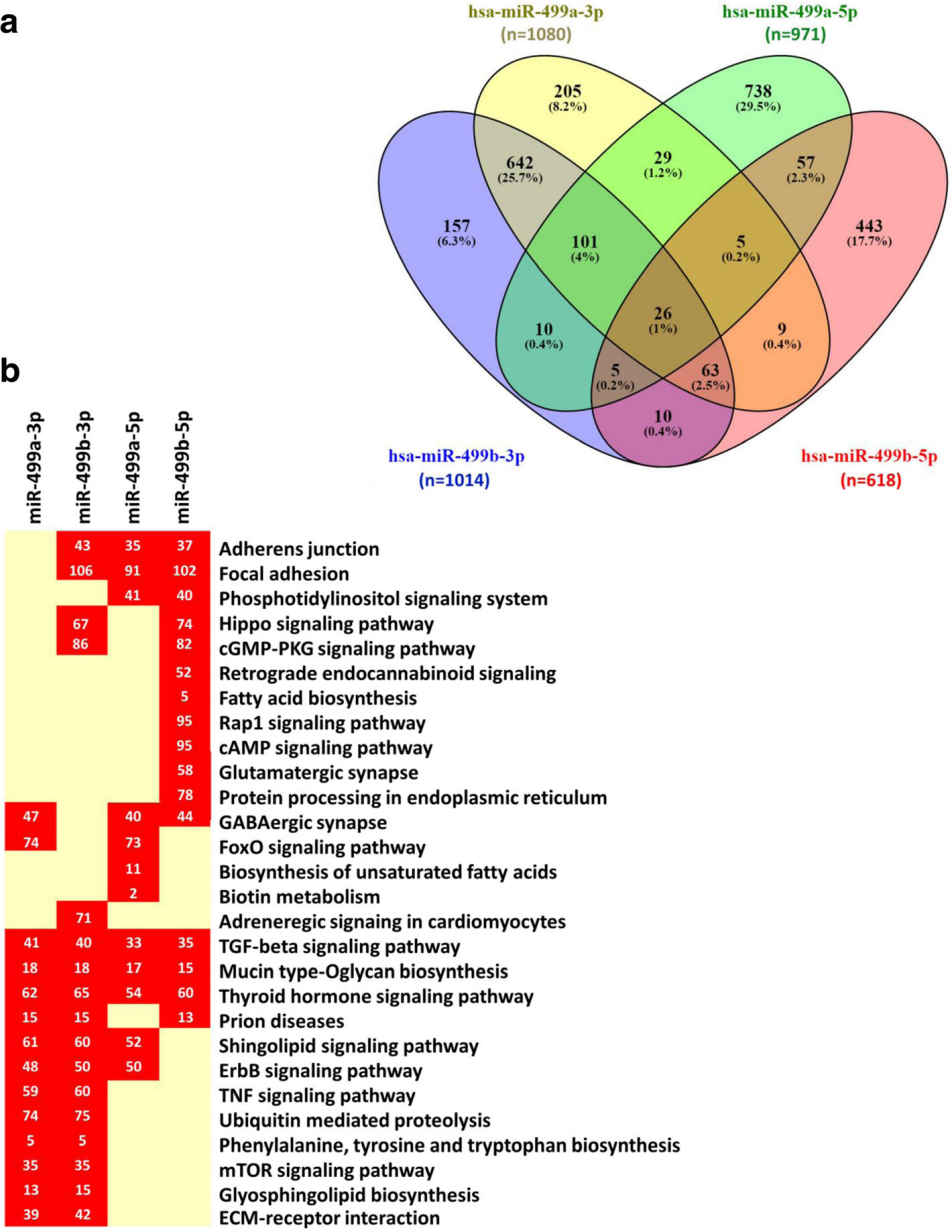
Enrichment pathway analysis of MIR-499 gene cluster targets. (**a**) Venn diagram showing the intersection of gene targets for each microRNA. Gene targets were retrieved from microT-CDS Diana tools (http://diana.imis.athena-innovation.gr/DianaTools/index.php?r=microT_CDS/index) with threshold set at 0.7 and significance <0.05. Venn diagram was drawn using Venny v2.0 (http://bioinfogp.cnb.csic.es/tools/venny/). The figure shows that hundreds of gene targets are complementary to miR-499a and miR-499b. Some of these genes are commonly targeted by both genes, showing some similarities in their function. However, other gene targets that are distinct for each mature form, indicate the presence of unique function for each of them. (**b**) Heat map showing top significant KEGG pathways involved by miRNA targets. Result intersection and filtration after FDR correction was applied. Fisher’s Exact test (hypergeometeric distribution) was used for statistical analysis. Heat map represent some of the significant KEGG pathways related to the target gene set of each mature miRNA. The number in the heat map represents the number of gene targets in each pathway affected by the corresponding miRNA. The four mature forms of MIR-499 gene family is involved in TGF-beta signaling pathway and mucin biosynthesis. Additionally, miR-499a-5p along with miR-499b are enrolled in adherence junction and focal adhesion

Hundreds of genes were predicted to be targeted by miR-499a and miR-499b using multiple microRNA databases. Though miRNAs form secondary hairpin loop, with complementary sequences in their structure; different gene sets were predicted to be targeted by both mature forms synthesized from either arm. A total of 1890 genes was predicted to be influenced by miR-499a (919 genes by 3p, 810 by 5p, and 161 genes by both). However, miR-499b was involved in manipulating the transcription of 1528 genes (910 genes by 3p, 514 by 5p, and 104 genes by both forms). Due to sequence similarity identified between miR-499a and miR-499b, their mature forms shared target genes; specifically 832 common genes are targeted by both miR-499a-3p and miR-499b-3p, whereas, 93 genes were the same for both 5p forms (Fig. 4a).

### Functional annotation clustering and pathway enrichment analysis

**Fig. 5:**
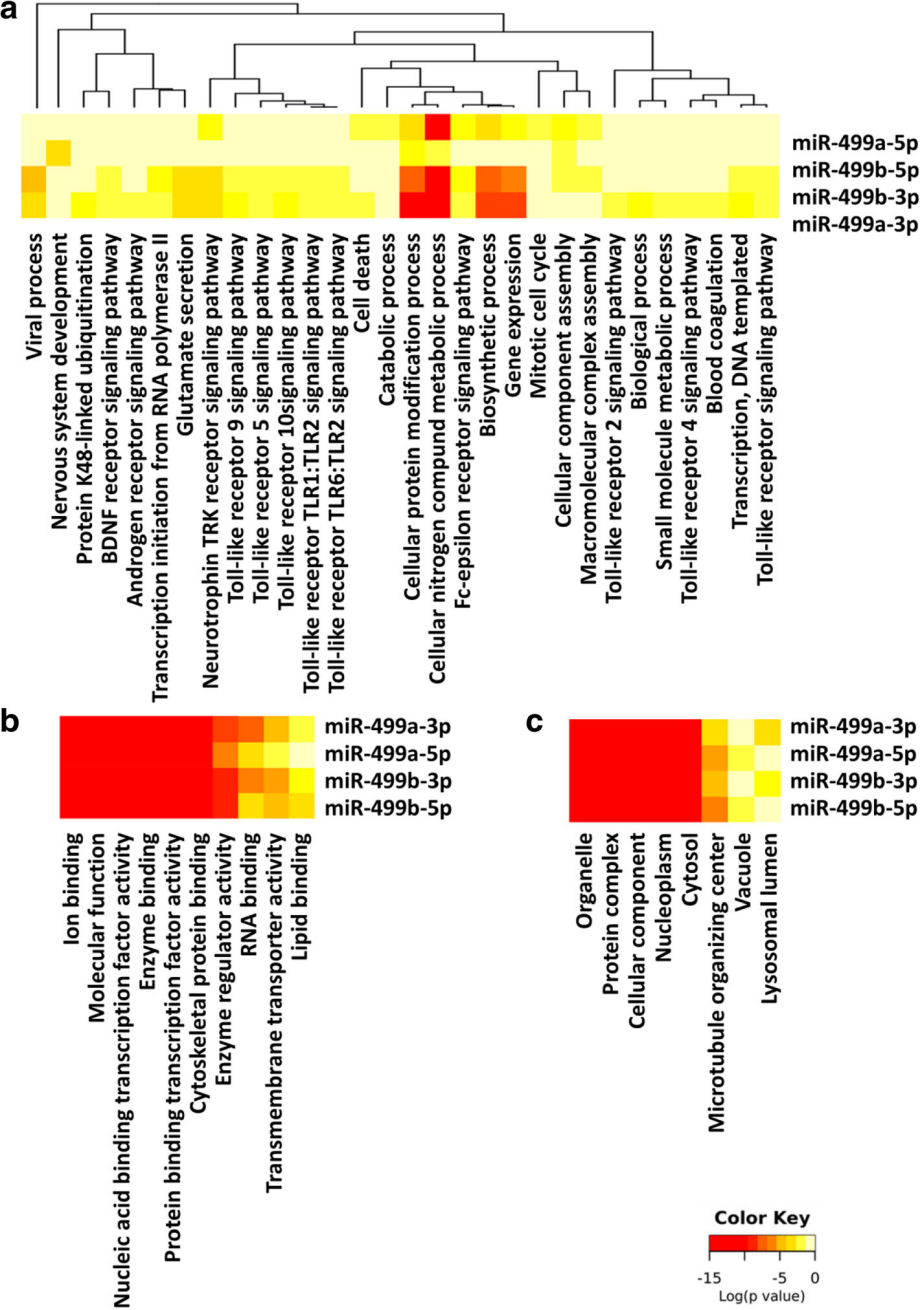
Functional annotation clustering of target genes of MIR-499 gene family. Enrichment pathway analysis. Heat map showing **a**) Biological process, (**b**) Molecular functions and (**c**) Cellular components for mature forms of both hsa-miR-499a and hsa-miR-499b with *p*-values <0.05 and microT-CDS threshold 0.5. Degree of color is based on the significant *p* values of the predicted algorithm by DIANA tools; the red has the highest score of statistical significance. The most pathogenomic feature shown in the heat maps is the involvement of miR-499 in different Toll-like receptor and Fc-epsilon receptor signaling pathways. Subsequent activation of these receptors release cytokines and mediators that drive immediate allergic airway reaction and contribute to intense eosinophil and lymphocyte infiltration in the airways

KEGG pathway enrichment analysis showed enrollment of miR-499 gene targets in remodeling and inflammation-related signaling pathways; including mucus biosynthesis and secretion, sphingolipid signaling, phosphatidylinositol signaling cell adhesion (focal adhesion and adherence junction pathways), fibrogenic and immune-modulator pathways (TGF beta signaling and TNF signaling pathways) (Fig. 4b). Functional clustering annotation of these gene sets identified the most significant their molecular activities, biological processes, pathways, and the cellular components where these genes execute their functions (Fig. 5). Comparison between the two microRNAs by miRpair 2GO explored the functional similarity scores for gene ontology to be 0.676 for biological process; 0.412 for molecular function, and 0.833 for cellular components. These targets were significantly clustered in four chromosomes; namely 67 genes on chromosome 3, 47 genes on chromosome 12, 31 genes on chromosome 16, and 30 genes on chromosome 19.

### Predicted functional effect of rs3746444 SNP

**Fig. 6:**
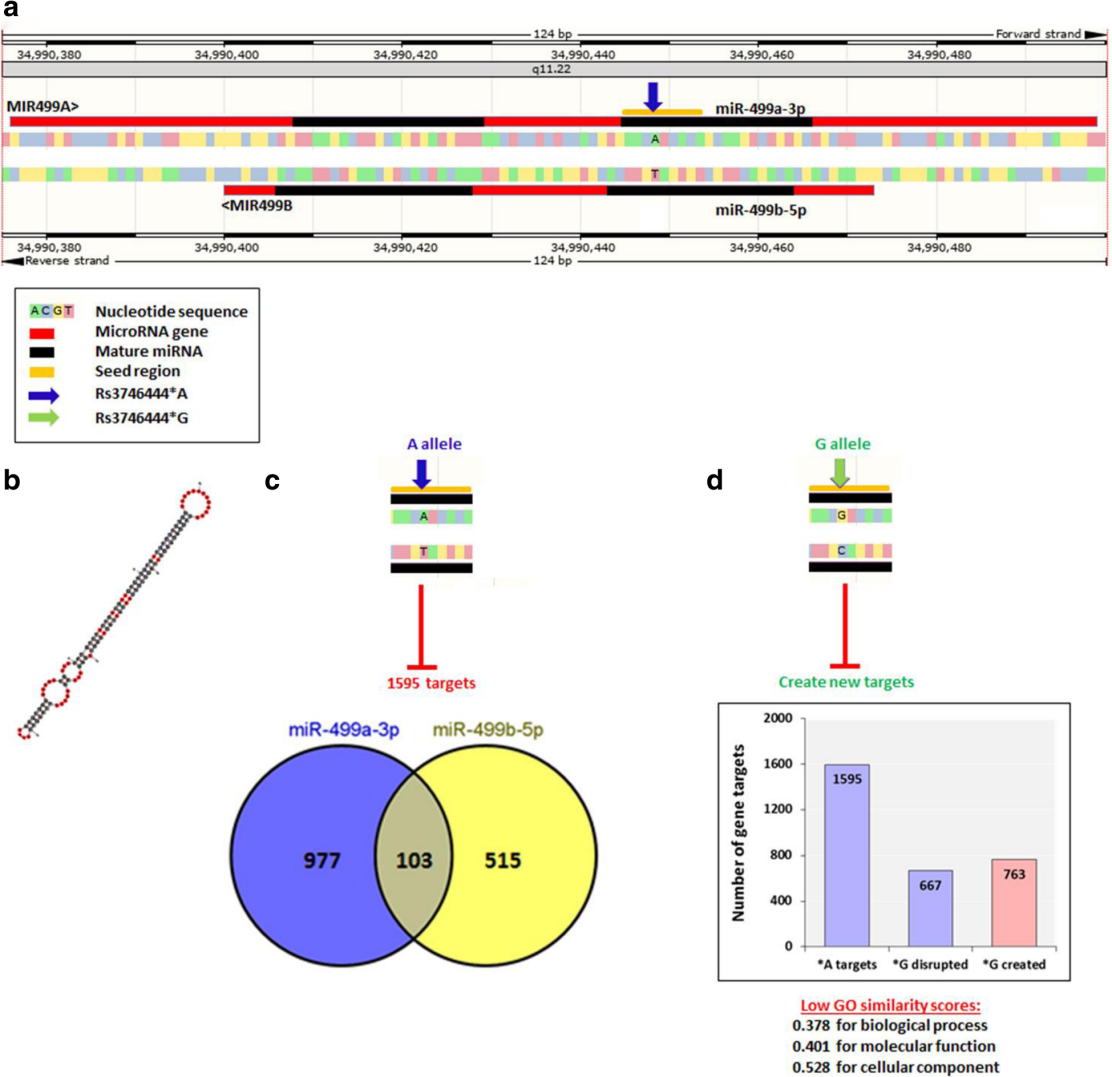
Structural and functional impact of rs3746444 variant in MIR-499A gene. **a**) Genomic location of the SNP on both strands. It represents the nucleotide number 73 (out of 122) of MIR499A on the forward strand (n.73A > G) within the sequence of miR-499a-3p and the nucleotide number 25 (out of 73) of MIR499B on the reverse strand (n.25 T > C) within the sequence of miR-499b-5p. (**b**) Predicted folding pattern of precursor stem loop structure by RNAfold and KineFold web-servers. No effect of the alleles on the folding pattern was detected. (**c**) Targets of A allele in miR-499a-3p and miR-499b-5p. (**d**) Predicted functional effects of G allele on gene targets and gene ontology. Being in the seed region at the 5′ end of miR-499a-3p generates the possibility of creating an altered target gene set for that particular. Using PolymiRTS Database 3.0 platform, identified the disruption of 667 (41.8%) genes of the miR-499a targets and creation of new 763 genes when A allele is substituted by G at the seed region sequence: AAC[A/G]UCACAGCAAGUCUGUGCU

In silico analysis identified the rs3746444 variant to overlap 3 genes. It lies in the intron of *MYH7B* gene (c.2103 + 138A > G in ENST00000262873 transcript or c.2100 + 138A > G in ENST00000618182 transcript). In addition, it represents the nucleotide number 73 (out of 122) of MIR499A on the forward strand (n.73A > G) within the sequence of miR-499a-3p and the nucleotide number 25 (out of 73) of MIR499B on the reverse strand (n.25 T > C) within the sequence of miR-499b-5p (Fig. 2d). Analyzing the secondary structure of pre-miR-499a hairpin loop with rs3746444 SNP (either A or G alleles) via RNAfold and KineFold web-servers showed no effect of the alleles on the folding pattern. The SNP does not overlap any regulatory region or motif features. However, being in the seed region at the 5′ end of miR-499a-3p generates the possibility of creating an altered target gene set for that particular. Using PolymiRTS Database 3.0 platform, identified the disruption of 667 (41.8%) genes of the miR-499a targets and creation of new 763 genes when A allele is substituted by G at the seed region sequence: AAC[A/G]UCACAGCAAGUCUGUGCU. Comparing the two gene sets of each allele by miRmut2Go web-server revealed low functional similarity scores for the three GO domains; 0.378 for biological process, 0.401 for molecular function, and 0.528 for cellular component similarity scores, (Fig. 6). Further enrichment analysis of the new gene list targeted by G allele was significantly involved in two KEGG pathways (glycan degradation and glycolysis/gluconeogenesis; *p* = 0.016 and 0.031 respectively), 45 genes of them were significantly located on a single chromosome (*p* = 0.044).

### Genotyping of rs3746444 polymorphism in the study groups

**Fig. 7:**
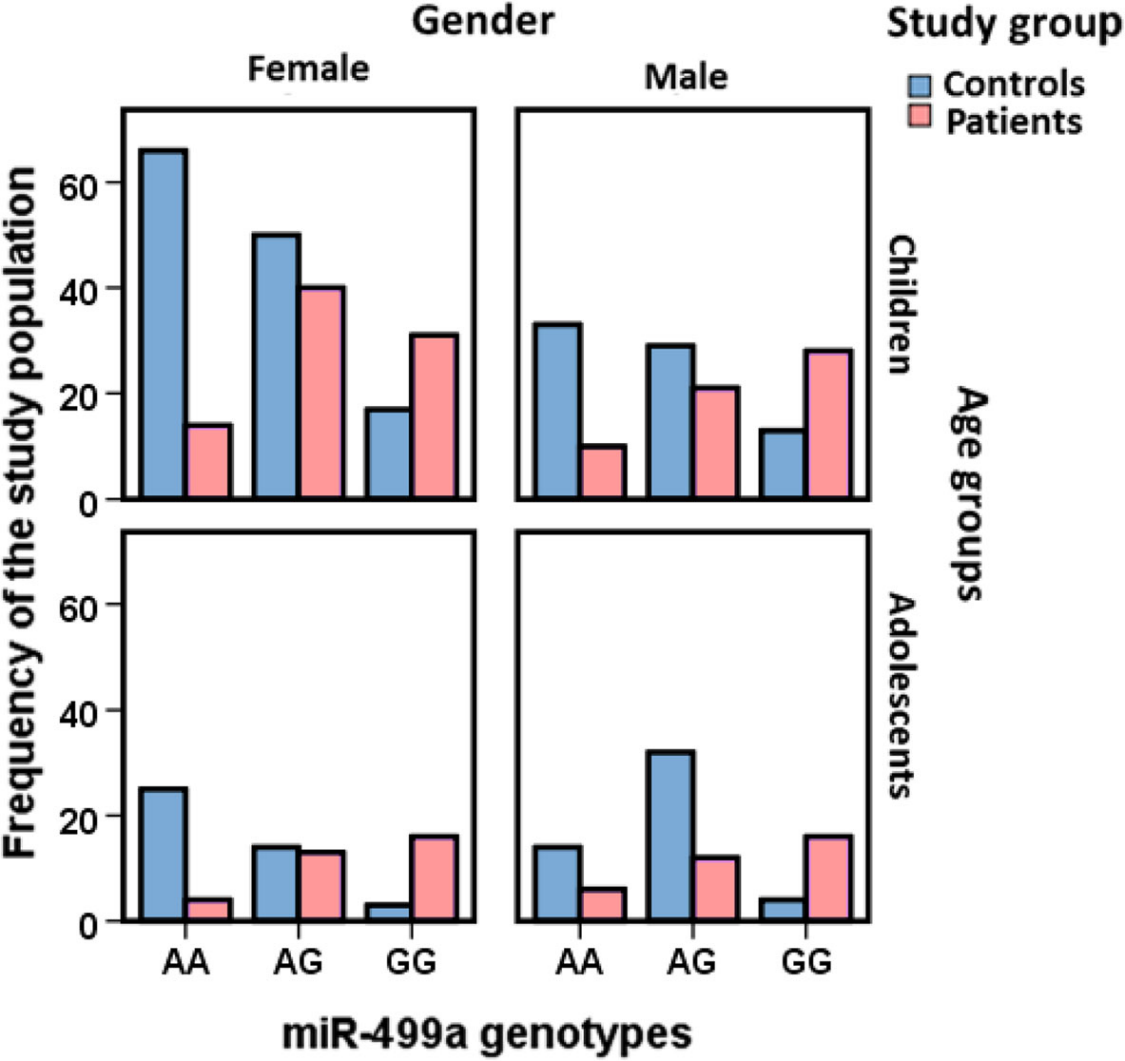
Allelic discrimination analysis of rs3746444 MIR-499A polymorphism in the study population. Genotype frequencies in patients and controls stratified by age and gender. AA genotype was more frequent in controls than their counterparts. Chi square test was used. Statistical significance at *p* < 0.05

Baseline characteristics of patients and control groups in both discovery and replication stages are illustrated in Additional file 1: Table S1. Genotype distribution among the studied groups was in agreement with Hardy Weinberg equilibrium (*p* > 0.05). Analysis of the whole study population (211 patients and 300 controls) revealed that the minor allele frequency (MAF; G allele) in the control group was 0.34; the same allele was doubled in asthmatic cohort, accounting for 0.63 (*p* < 0.001). Correspondingly, there was higher frequency of GG genotype among asthmatic patients (*p* < 0.001), Table 1. Both discovery and replication cohorts demonstrated similar trends in genotype and allele frequencies. Genetic association model analysis showed that individuals with G variant were more likely to develop asthma than non-carriers under all association models (G versus A: OR = 3.27, 95% CI = 2.53–4.22; GG versus AA: OR = 9.52, 95% CI = 5.61–16.5; AG versus AA: OR = 2.13, 95% CI = 1.24–3.46; GG + AG versus AA: OR = 4.43, 95% CI = 2.88–6.82), Table 1. High significant proportions of AG and GG genotypes were also observed in asthmatic children and adolescents of both gender (*p* < 0.001) (Fig. 7).

**Table 1:** Genotype and allele frequencies of hsa-miR-499a seed region variant in asthma patients and controls

		Asthma	Controls	*P* value	Crude OR (95%CI)	Adjusted OR (95%CI) ^a^
DISCOVERY STAGE						
Total number		(*n* = 96)	(*n* = 96)			
Genotype frequency						
*Co-Dominant model*	AA	20 (20.8)	56 (58.3)	**<0.001**	1.0	1.0
	AG	38 (39.6)	30 (31.3)		**3.54 (1.76–7.14)**	**3.67 (1.74–7.77)**
	GG	38 (39.6)	10 (10.4)		**10.6 (4.48–25.2)**	**10.9 (4.41–27.3)**
*Dominant model*	AA	20 (20.8)	56 (58.3)		1.0	
	AG + GG	76 (79.2)	40 (41.7)		**5.32 (2.81–10.1)**	
P-HWE		0.064	0.078			
Allele frequency						
*Allelic model*	A	78 (40.6)	142 (73.9)	**<0.001**	1.0	
	G	114 (59.3)	50 (26.04)		**4.1 (2.69–6.39)**	
Carriage rate						
	A	58 (60.4)	86 (89.6)	**<0.001**	**0.17 (0.08–0.38)**	**0.29 (0.12–0.70)**
	G	76 (79.2)	40 (41.7)	**<0.001**	**4.71 (2.52–8.80)**	**3.22 (1.57–6.62)**
REPLICATION STAGE						
Total number		(*n* = 115)	(*n* = 204)			
Genotype frequency						
*Co-Dominant model*	AA	14 (12.2)	82 (40.2)	**<0.001**	1.0	1.0
	AG	48 (41.7)	95 (46.6)		**2.95 (1.52–5.75)**	**2.79 (1.46–5.06)**
	GG	53 (46.1)	27 (13.2)		**11.4 (5.52–23.9)**	**10.8 (4.65–20.3)**
*Dominant model*	AA	14 (12.2)	82 (40.2)	**<0.001**	1.0	
	AG + GG	101 (87.8)	122 (59.8)		**4.84 (2.59–9.06)**	
P-HWE		0.542	0.950			
Allele frequency						
*Allelic model*	A	76 (33.1)	259 (63.5)	**<0.001**	1.0	
	G	154 (66.9)	149 (36.5)		**3.52 (2.50–4.95)**	
Carriage rate						
	A	62 (53.9)	177 (86.8)	**<0.001**	**0.17 (0.1–0.30)**	**0.23 (0.2–0.34)**
	G	101 (87.8)	122 (59.8)	**<0.001**	**4.84 (2.59–9.06)**	**4.79 (2.64–8.9)**
OVERALL ANALYSIS						
Total number		(*n* = 211)	(*n* = 300)			
Genotype frequency						
*Co-Dominant model*	AA	34 (16.1)	138 (46.0)	**<0.001**	1.0	1.0
	AG	86 (40.8)	155 (41.7)		**2.25 (1.42–3.56)**	**2.13 (1.24–3.46)**
	GG	91 (43.1)	37 (12.3)		**9.98 (5.84–17.05)**	**9.52 (5.61–16.5)**
*Dominant model*	AA	34 (16.1)	138 (46.0)			
	AG + GG	177 (83.9)	162 (54.0)	**<0.001**	1.0	
P-HWE		0.079	0.507		**4.43 (2.88–6.82)**	
Allele frequency						
*Allelic model*	A	154 (36.5)	431 (62.3)	**<0.001**	1.0	
	G	268 (63.5)	229 (34.7)		**3.27 (2.53–4.22)**	
Carriage rate						
	A	120 (56.9)	263 (87.7)	**<0.001**	**0.18 (0.12–0.28)**	**0.12 (0.08–0.21)**
	G	175 (82.9)	162 (54.0)	**<0.001**	**4.14 (2.71–6.33)**	**3.96 (2.14–5.9e)**

Values are presented as number (percentage). P-HWE, *p* values of Hardy-Weinberg equilibrium

^a^Adjusted for confounding variables (age, gender, residence, family history, BMI percentile, puberty) was done by binary logistic regression (Method = Enter). Chi-square test was used. Bold values indicate significance  at *p* < 0.05

**Fig. 8:**
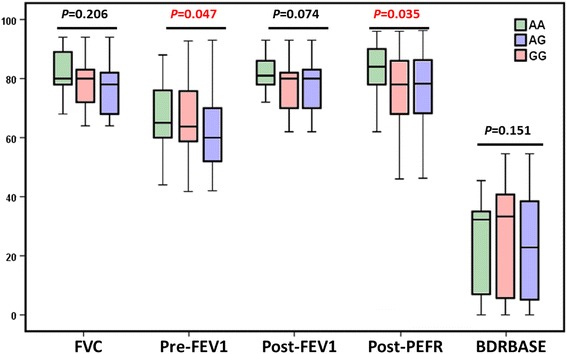
Bronchodilator response in asthmatic patients, according to MIR-499a genotypes. Pre-FEV1: pre-bronchodilator forced expiratory volume in one second before salbutamol inhalation, post-FEV1: post-bronchodilator forced expiratory volume in one second after salbutamol inhalation, post-PEFR: post-peaked expiratory flow rate, BDRBASE: change in FEV1 as a percent of baseline FEV1 [BDRBASE = ((postbronchodilator FEV1 − prebronchodilator FEV1) / prebronchodilator FEV1) × 100]

Genotype distribution according to disease characteristics of patients is shown in Table 2. There was no significant association of MIR-499a genotypes with any clinical or laboratory characteristics in the overall and stratified analysis. However, children and adolescents with early age at onset of asthma disease had higher frequency of GG genotype (*p* = 0.036). Additionally, GG homozygote patients have the lowest pre-bronchodilator FEV_1_ (*p* = 0.047) and the worst bronchodilator response after Salbutamol inhalation represented in low peaked expiratory flow rate (PEFR) (*p* = 0.035) (Fig. 8). Similarly, data exploration by multivariate analysis did not demonstrate clustering of asthmatic patients according to their genotypes or any other clinical variables (Additional file 2: Figure S1).

**Table 2:** Clinical and laboratory data of asthmatic patients, according to miR-499A rs3746444 genotypes

Characteristics	No	rs3746444 genotypes			*P* value
		AA	AG	GG	
Total number (%)	211	34 (16.1)	86 (40.8)	91 (43.1)	
Mean age, years					
Children (6–11)	144	24 (16.7)	61 (42.4)	59 (41.0)	0.650
Adolescents (12–18)	67	10 (14.9)	25 (37.3)	32 (47.8)	
Gender					
Female	118	18 (15.3)	53 (44.9)	47 (39.8)	0.380
Male	93	16 (17.2)	33 (35.5)	44 (47.3)	
FH of asthma	56	8 (14.3)	21 (37.5)	27 (48.2)	0.666
Pubertal status	109	19 (17.4)	46 (42.2)	44 (40.4)	0.685
Obesity	23	3 (13.0)	11 (47.8)	9 (39.2)	0.838
Residence					
Urban	75	12 (16.0)	29 (38.7)	34 (45.3)	0.879
Rural	136	22 (16.2)	57 (41.9)	57 (41.9)	
Mean age at onset, years		3.82 ± 1.6	3.58 ± 1.9	3.25 ± 1.8	0.245
Early (≤3y)	99	10 (10.1)	39 (39.4)	50 (50.5)	**0.036**
Late (>3y)	112	24 (21.4)	47 (42.0)	41 (36.6)	
Asthma duration, years		5.55 ± 2.5	5.96 ± 2.3	6.23 ± 2.5	0.245
Asthma phenotypes					
Atopic asthma	166	28 (16.8)	66 (39.8)	72 (43.4)	0.611
Non-atopic asthma	15	4 (26.7)	5 (33.3)	6 (40.0)	
Aspirin-sensitive asthma	25	1 (4.0)	13 (52.0)	11 (44.0)	
Exercise-induced asthma	5	1 (20.0)	2 (40.0)	2 (40.0)	
Symptoms					
Daytime symptoms >2/week	103	14 (13.6)	42 (40.8)	47 (45.6)	0.581
Nocturnal symptoms	39	6 (15.4)	18 (46.2)	15 (38.6)	0.741
Reliever use >2/week	74	14 (189)	35 (47.3)	25 (33.8)	0.131
Activity limitations	69	11 (15.9)	28 (40.6)	30 (43.5)	0.997
Asthma control					
Well controlled	76	16 (21.1)	28 (36.8)	32 (42.1)	0.122
Partly controlled	99	9 (9.1)	44 (44.4)	46 (46.5)	
Uncontrolled	36	9 (25.0)	14 (38.9)	13 (36.1)	
Asthma severity					
Mild	88	15 (17.0)	35 (39.8)	38 (43.2)	0.506
Moderate	90	15 (16.7)	33 (36.7)	42 (467)	
Severe	33	4 (12.1)	18 (54.5)	11 (33.3)	
Co-morbidities	105	17 (16.2)	43 (41.0)	45 (42.8)	0.997
Airway hyper-responsiveness					
Normal	61	11 (18.0)	20 (32.8)	30 (49.2)	0.127
Borderline	61	13 (21.3)	31 (50.8)	17 (27.9)	
Mild/moderate	54	7 (13.0)	21 (38.9)	26 (48.1)	
Severe	35	3 (8.6)	14 (40.0)	18 (51.4)	
Lab tests					
Total IgE (IU/ml)	211	60 (42–121)	162 (15–122)	45 (15–120)	0.307
AEC (×10^6^/L)	211	111 (31.5–340)	88 (23.7–245)	65 (25–230)	0.571

Values are shown as mean ± standard deviation, median (quartiles) or as a number (percentage). AEC; Absolute eosinophilic count, FH; family history. Assessment and classifications of asthma were performed based on GINA guidelines [23]. Chi-square, ANOVA, and Kruskal-Wallis tests were used. Bold values indicate significance at *p* < 0.05

## Discussion

MicroRNAs are involved in various biological cellular processes. Aberrant transcriptomic signatures of various miRNAs have been detected in the airways and circulation of asthmatic patients [33]. Sequence variations within miRNA genes, especially in the mature miRNA seed region, may have a profound impact on miRNA biogenesis and function [34, 35]. In 2009, an inflammation-related MIR-499A was identified within the intronic sequence of the myosin *MYH7B* gene [36]. Several lines of evidence suggest that miR-499a play critical roles in orchestrating the immune response in various human disorders [11, 12, 37]. Bioinformatics analysis determined complement base pairing with important cytokines as IL-13 and IL-23, both are recognized effectors in asthma pathway. IL-13 signaling results in mucin hypersecretion, airway remodeling and fibrosis, and bronchial hyper-responsiveness [38]. IL-23 induction regulates allergic asthma through modulation of TH2 cell differentiation and eosinophilic infiltration [39]. Functional enrichment clustering illustrated the involvement of miR-499a-3a and miR-499a-3b in Toll-like receptor (TLC) signaling pathways that are implicated in the initial phase of the host defense against invading pathogens and allergens leading to TH2 activation [40, 41]. In addition, miR-499a and miR-499b are involved in Fc-epsilon signaling pathway; cross-linking of IgE with FcεRI receptors on mast cells, causes the release of leukotrienes and histamines essential for immediate allergic reaction. Other biological processes as gene expression, cellular protein modification process, transcription initiation from RNA polymerase II, and cellular component assembly are significantly mediated by miR-499 gene family.

Using various computational tools, miR-499a and miR499b were predicted to target key molecules in asthma-related KEGG pathways. They could modulate 18 targets in mucin type O-biosynthesis pathway related to polypeptide N-Acetylgalactosaminyltransferase (GALNT) genes. Hyperglycosylated mucins are commonly found in chronic inflammatory airway diseases such as asthma, for which inhibition of mucin and their glycosylation could contribute in controlling the disease [42]. The next most significant pathway was fatty acid biosynthesis with 5 target genes. Recent studies demonstrated the vital role of fatty acids in the formation of inflammatory mediators relevant to the pathophysiology of asthma [43]. Another identified pathway that is highly impacted by miR-499 gene family is glycosphingolipid (GSL) biosynthesis. β-Glycosphingolipids have emerged as a family of potential ligands for natural killer (NK) cells. Extensive infiltration of NK cells in the airway bronchial mucosa is considered one of the prominent driver in asthma development via inducing the secretion of TH2 cytokines that results in airway hyper-responsiveness and inflammation [44]. Lowering GSL levels in mast cells in an animal model of asthma was found to ameliorate disease manifestations [45]. Both mature forms of miR-499a and miR-499b were predicted to target about 40 and 60 genes in TGF-beta and TNF signaling pathways, important mediators contributing in lung tissue fibrosis and proliferation of smooth muscle cells with subsequent airway remodeling [46]. We also identified several targets for miR-499 gene family in adherence junction and focal adhesion pathways. Tight junctions not only seal the pulmonary epithelium and maintain the structural integrity of the airway walls, but also act as key regulators for epithelial cell proliferation and differentiation. Dysfunction of this homeostasis could participate in airway wall remodeling in asthma disease [47].

Within MIR499A and MIR499B genes, bioinformatics analysis revealed the presence of a common polymorphism at the position 20:34,990,448 within the seed region of mature miR-499a-3p (rs3746444; AC[A/G]UCAC). In the current study, we genotyped rs3746444 polymorphism in asthmatic children and adolescents compared to controls. Genotype frequencies did not deviate from the Hardy-Weinberg equilibrium in the study groups. G variant was associated with increased susceptibility to develop bronchial asthma under all genetic association models. Despite the MAF (G) represented 0.26 in the control group, it accounted for 0.59 among patients. The same allele was found to be associated with poor pre-bronchodilator FEV_1_ in asthmatic patients. In addition, homozygotes for G had the worst bronchodilator response after Salbutamol inhalation. That was consistent with prior studies; G allele was also associated with increased risk of developing of several autoimmune and inflammatory diseases. miR-499a*G carriers showed higher susceptibility to RA in Egyptians [12] and Iranian people [48], Behcet’s disease in Turks [15], ulcerative colitis in Japanese [49], ankylosing spondylitis [16], idiopathic recurrent spontaneous abortion in Koreans [50], and coronary artery disease [51, 52]. In addition, individuals with rs3746444*G allele showed a more active phenotype of RA in Egyptian female patients [53] and a severe course of multiple sclerosis [54].

Though our in silico data analysis revealed that the substitution of A > G has no dramatic effect on the folding pattern of the hairpin loop structure, each allele was predicted to have different set of gene targets. A total of 1890 genes is influenced by mature miR-499a with rs3746444*A variant. In contrast, rs3746444*G allele could delete 41.8% of these genes and create new 763 targets. By conducting functional annotation clustering and enrichment analysis, our results illustrated that gene sets for each allele only shared the same 37.8%, 40.1%, and 52.8% for biological process, molecular function, and cellular components, respectively. In particular, G allele targets were significantly involved in two new KEGG pathways (other glycan degradation and glycolysis/gluconeogenesis). In the former pathway, miR-499 can target both aspartylglucosaminidase (*AGA*) gene involved in the catabolism of N-linked oligosaccharides of glycoproteins in the lysosomes, and galactosidase beta 1 (*GLB1*) gene, which plays functional roles in the formation of extracellular elastic fibers and in the development of connective tissue. In the other pathway for glycolysis/gluconeogenesis, miR-499 mainly target aldehyde dehydrogenase 1 family member A3 (*ALDH1A3*) and alcohol dehydrogenase 1 beta polypeptide (*ADH1B*) genes, members of the enzyme family which metabolize a wide variety of substrates as multiple inositol-polyphosphate phosphatase 1, an important second messenger in eukaryotic cells. *ALDH1A3* gene was found to be over-expressed more than two times in asthmatic patients and 3.5 times during exacerbations [55]. Further, in vivo functional analysis is warranted to explore the mechanistic regulation of these targets in the etiopathogenesis of asthma.

In some previous studies, computational methods were devised to study the effects of air-borne dust through high fidelity computational simulation of different particle sizes over several breathing cycles [56], modeling lung pressures [57], and developing 3D Euler Lagrangian models to obtain the regional deposition of the poly-dispersed drug Budesonide [58]. It would be a beneficial next step to assess the pharmacogenomics effect of variants with the regional deposition of dust particles and drug delivery to the respiratory tract during respiration.

To the best of our knowledge, this is the first study highlighting the role of miR-499 rs3746444 in bronchial asthma disease. However, some limitations need to be addressed. First, the small relative sample size in the current study might underestimate the synergistic effect of miR-499 SNP with environmental exposure. Second, the impact of the SNP was not correlated with the miRNA expression profile. Third, functional assessment of targets involved in bronchodilator response and methacholine challenge test is mandatory to develop more efficient therapeutic strategies.

## Conclusions

In conclusion, our study suggested that the rs3746444 (A > G) polymorphism in miR-499 gene family might contribute to the susceptibility of asthma in children and adolescents with bronchial asthma. Additional studies, including larger cohorts with diverse ethnic background, and functional tests are warranted to explore the immunomodulatory mechanism of miR-499 genes in bronchial asthma for developing more specific theranostic agents.

## Additional files

Additional file 1: Table S1. Baseline characteristics of the study groups. **Table S2.**. Predicted target gene sets for mature miR-499a and miR-499b. (http://www.microrna.gr/microT-CDS). (ZIP 79 kb)

Additional file 2: Figure S1. Principal components analysis of asthmatic patients. Ordination plot constructed using 211 patients strand and 20 clinical and laboratory variables. Samples are distributed along two axes. Axis 1 explains 22.6% of variance among patients, whereas axis 2 resolves 11.3% of variance. Samples are scattered and colored according to their genotype; AA (red), AG (green), and GG (blue). PCA did not reveal clustering of patients according to their genotypes. (DOCX 95 kb)
