# Supplementary material for: Structure and functional impact of seed region variant in MIR-499 gene family in bronchial asthma
Source: Respir Res. 2017 Sep 8;18:169. doi: 10.1186/s12931-017-0648-0 (PMC5591547; doi:10.1186/s12931-017-0648-0)
Supplement: Supplementary file 1 — Baseline characteristics of the study groups. Table S2.. Predicted target gene sets for mature miR-499a and miR-499b. (http://www.microrna.gr/microT-CDS). (ZIP 79 kb) [file 12931_2017_648_MOESM1_ESM.zip › Suppl. Table S1.docx]

| **Supplementary Table S1. Baseline characteristics of the study groups.** | | | | | | | | | |
| --- | --- | --- | --- | --- | --- | --- | --- | --- | --- |
| **Demographic data** | | **Discovery cohorts** | | **P values** | **OR (95%CI)** | **Replication cohorts** | | **P values** | **OR (95%CI)** |
|  |  | **Asthma** | **Controls** |  |  | **Asthma** | **Controls** |  |  |
| **Total number** |  | 96 (100) | 96 (100) |  |  | 215 (100) | 204 (100) |  |  |
| **Mean age**, years |  | 9.7 ± 3.0 | 9.6 ± 3.1 | 0.761 |  | 10.2 ± 3.1 | 10.3±3.3 | 0.872 |  |
| **Age categories**, % | 6-11 | 72 (75.0) | 73 (76.0) | 0.867 | *Reference* | 75 (62.6) | 135 (66.2) | 0.543 | *Reference* |
|  | 12-18 | 24 (25.0) | 23 (24.0) |  | 1.05 (0.54-2.04) | 43 (37.4) | 69 (33.8) |  | 1.16 (0.72-1.88) |
| **Gender** | Male | 44 (54.2) | 40 (41.7) | 0.561 | *Reference* | 66 (57.4) | 119 (58.3) | 0.906 | *Reference* |
|  | Female | 52 (45.8) | 56 (58.3) |  | 1.18 (0.66-2.09) | 49 (42.6) | 85 (41.7) |  | 1.03 (0.65-1.65) |
| **Residence** | Urban | 32 (33.3) | 45 (46.9) | 0.056 | *Reference* | 43 (37.4) | 91 (44.6) | 0.238 | *Reference* |
|  | Rural | 64 (66.7) | 51 (53.1) |  | 0.56 (0.31-1.01) | 72 (62.6) | 113 (55.4) |  | **2.07 (1.3-3.31)** |
| **BMI percentile** | <85^th^ | 53 (55.2) | 51 (53.1) | 0.952 | *Reference* | 62 (53.9) | 129 (63.2) | 0.197 | *Reference* |
|  | <95^th^ | 32 (33.3) | 34 (35.4) |  | 0.90 (0.48-1.67) | 41 (35.7) | 62 (30.4) |  | 1.37 (0.83-2.26) |
|  | ≥95^th^ | 11 (11.5) | 11 (11.5) |  | 0.96 (0.38-2.41) | 12 (10.4) | 13 (6.4) |  | 1.92 (0.82-4.45) |
| **Pubertal status** | Positive | 45 (46.8) | 44 (45.8) |  |  | 62 (53.9) | 112 (54.9) |  |  |
|  | Males | 25 (26.0) | 19 (19.8) | 0.243 | *Reference* | 28 (24.3) | 38 (18.6) | 0.143 | *Reference* |
|  | Females | 20 (20.8) | 25 (26.0) |  | 0.60 (0.26-1.40) | 34 (29.5) | 74 (36.2) |  | 0.62 (0.33-117) |
| **FH of asthma** | Negative | 67 (69.8) | 89 (92.7) | **<0.001** | *Reference* | 88 (76.5) | 174 (85.3) | 0.067 | *Reference* |
|  | Positive | 29 (30.2) | 7 (7.3) |  | **5.50 (2.27-13.3)** | 27 (23.5) | 30 (14.7) |  | 1.78 (0.99-3.17) |
| Values are presented as number (percentage) and mean ± standard deviation. BMI; body mass index, pubertal status; sexual maturation, and secondary sexual characters were assessed using Sexual Maturity Rating stages by Marshall and Tanner [25], FH; family history. Fisher's Exact, Chi-square and Student-t tests were used. Bold values are statistically significant at *p* < 0.05. | | | | | | | | | |
