# Supplementary material for: Structure and functional impact of seed region variant in MIR-499 gene family in bronchial asthma
Source: Respir Res. 2017 Sep 8;18:169. doi: 10.1186/s12931-017-0648-0 (PMC5591547; doi:10.1186/s12931-017-0648-0)
Supplement: Supplementary file 2 — Principal components analysis of asthmatic patients. Ordination plot constructed using 211 patients strand and 20 clinical and laboratory variables. Samples are distributed along two axes. Axis 1 explains 22.6% of variance among patients, whereas axis 2 resolves 11.3% of variance. Samples are scattered and colored according to their genotype; AA (red), AG (green), and GG (blue). PCA did not reveal clustering of patients according to their genotypes. (DOCX 95 kb) [file 12931_2017_648_MOESM2_ESM.docx]

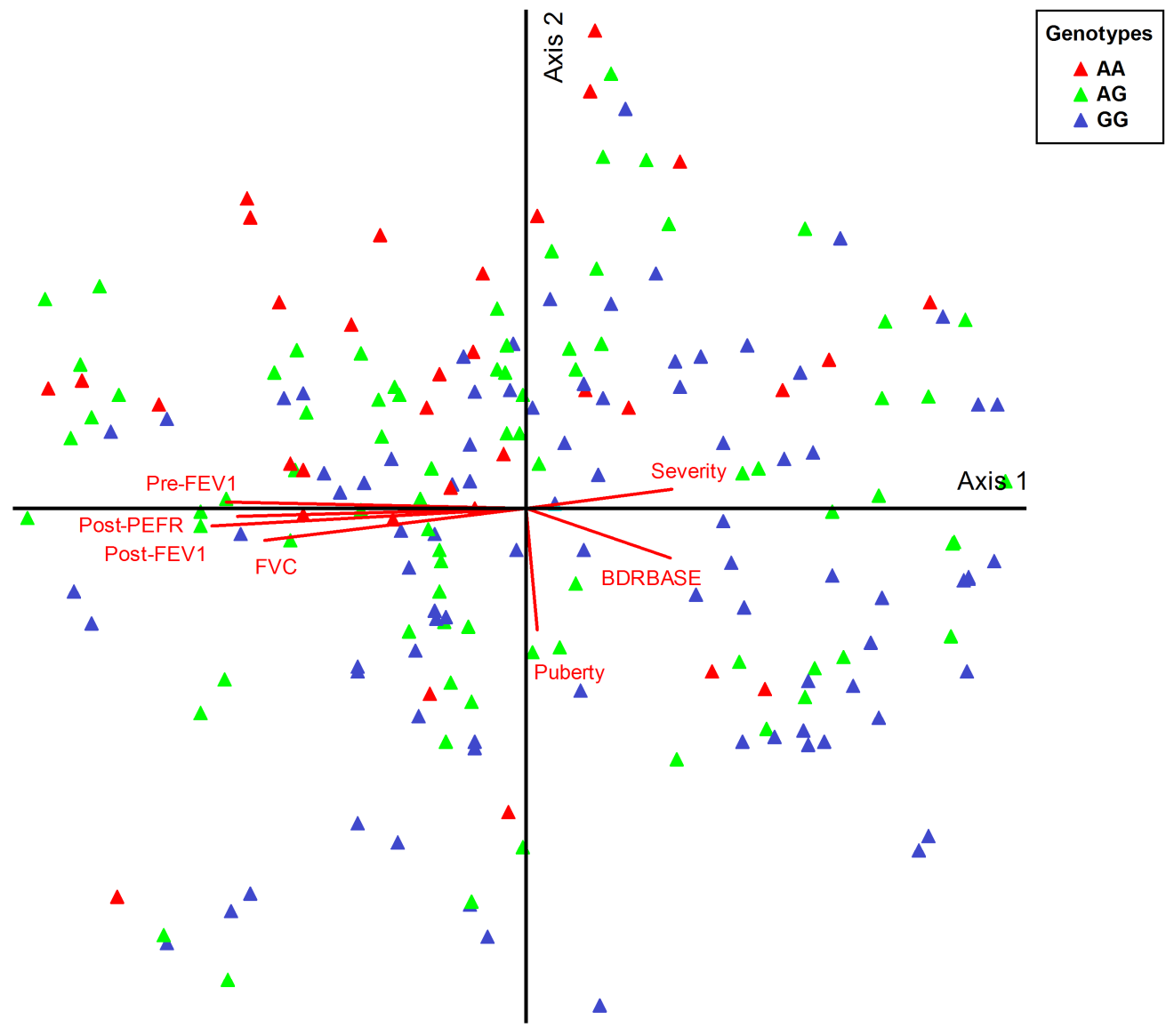


**Figure S1. Principal components analysis of asthmatic patients.** Ordination plot constructed using 211 patients strand and 20 clinical and laboratory variables. Samples are distributed along two axes. Axis 1 explains 22.6% of variance among patients, whereas axis 2 resolves 11.3% of variance. Samples are scattered and colored according to their genotype; AA (red), AG (green), and GG (blue). PCA did not reveal clustering of patients according to their genotypes.
